# The Relationship Between Big Five and Self-Control in Boxers: A Mediating Model

**DOI:** 10.3389/fpsyg.2019.01690

**Published:** 2019-08-08

**Authors:** Guodong Zhang, Xin Chen, Luxia Xiao, Yun Li, Bing Li, Zi Yan, Liya Guo, Detlef H. Rost

**Affiliations:** ^1^Key Lab of Physical Fitness Evaluation and Motor Function Monitoring of General Administration of Sports of China, College of Physical Education, Institute of Sports Science, Southwest University, Chongqing, China; ^2^Center for Mental Health Education, School of Psychology, Southwest University, Chongqing, China; ^3^Health Sciences Department, Merrimack College, North Andover, MA, United States; ^4^Department of Child and Youth Psychology, Faculty of Psychology, Philipps-University Marburg, Marburg, Germany

**Keywords:** Big Five personality traits, self-efficacy, self-control, mediating effect, boxer

## Abstract

Self-control seems to be the core element for achieving optimal competitive performance, and be of great importance to well-being and healthy development of humans. According to the literature, there exist some correlations between personality traits and self-control. The aim of this study was to shed some additional light on the relationship between the Big Five personality traits and self-control in boxers and investigate self-efficacy as a mediator between the two variables. Two hundred and ten boxers (age: *M* = 18.89, SD = 3.83; amount of boxing practice: *M =* 4.93 years, SD = 3.22; 76 males) of Chinese national athletes participated the study. Results showed a pronounced level of self-control. The higher the competitive level, the higher the level of self-control. There were significant correlations among the Big Five, self-control, and self-efficacy. Self-efficacy mediated the relationship between the Big Five personality traits and self-control.

## Introduction

Self-control refers to the phenomenon of people overcoming their natural and automatic tendencies, desires, and behaviors and resisting short-term temptations to achieve long-term goals (Baumeister et al., 2007). It is essential for the well-being and healthy development of humans. Sigmund Freud believed that self-control is a major characteristic of a civilized society (Freud, 1993), while Hare et al. suggested that “the ability to exercise self-control is the key to human success and happiness” (Hare et al., 2009). Good self-control not only prevents drug abuse, criminal offenses, and other undesirable social behaviors, but also promotes the healthy growth of individuals and the harmonious development of society (Moffitt et al., 2011; Tangney et al., 2014). Self-control seems to be the core element for achieving optimal competitive performance (Zhang, 2013; Englert, 2016). Scholars in sport psychology have called for research that “gives a voice” to marginalized groups, which would arguably include boxers (Fisher et al., 2003; Ryba and Wright, 2005; Simpson and Wrisberg, 2013). Boxers should have a pronounced self-control and self-efficacy (Lane, 2006; Yu et al., 2012). Thus, boxing is well suited for applied sport psychology interventions (Schinke, 2007); more emphasis should be put into research on the self-control and self-efficacy of boxers.

Exploring the relationships between self-control, personality traits, and self-efficacy can serve as a starting point for an in-depth study of self-control. Mao et al. (2018) found that openness, conscientiousness, extraversion, and agreeableness were all positively correlated with self-control, and neuroticism was negatively related to self-control. In addition, Vera et al. (2004) believe that self-control is affected by self-efficacy. Most of the existing research on self-control in sporting contexts focused on soccer players, divers, middle-distance runners, and college athletes of all types. There is only limited research on the psychology of boxing in China, especially on the relationship between boxers’ personality traits and self-control. This study invited Chinese boxers of national athletes to take part in an examination of the relationship between their personality traits and self-control, and to look for any mediating effect of self-efficacy.

### Personality Traits and Self-Control

Previous studies have well documented the relationship between the Big Five and self-control. Neuroticism has a negative correlation with self-control; agreeableness, extraversion, openness, and responsibility are positively related to self-control; self-control is a prerequisite for individuals to adapt to their social environment (Andrei et al., 2014; Coyne and Wright, 2014). In addition, researchers found differences in the relationship between extraversion, openness, and self-control; some studies found that extraversion and self-control had a significant negative correlation (Green et al., 2016), while some others found extraversion was not significantly related with self-control (De Vries and Van Gelder, 2013). In the same way, researches on the relationship between openness and self-control had also produced inconsistent results (Andrei et al., 2014; Bazzy et al., 2017). Due to its consistency and stability across languages and cultures (Pilarska, 2018), the Big Five is often used to predict self-control (McCrae and Terracciano, 2005). Therefore, due to the inconsistency of the relationship between Big Five and self-control, and lack of studies among boxers, we hypothesized that 1: Neuroticism is negatively correlated with self-control, while agreeableness, conscientiousness, and extraversion are positively correlated with self-control among Chinese boxers.

### Personality Traits and Self-Efficacy

Personality traits are important factors influencing the self-efficacy of individuals (Stajkovic et al., 2018). Studies have linked the Big Five traits and self-efficacy (Judge et al., 2007; Cristina et al., 2018; Coco et al., 2019), for example, neuroticism is negatively correlated with self-efficacy, and extraversion, openness, agreeableness, and responsibility positively correlated with self-efficacy (Judge et al., 2007). Some scholars found that individuals with higher scores of conscientiousness had higher self-efficacy beliefs (Brown et al., 2011). Openness shifts perceptions of demands into challenges to be tackled, broadening task engagement and self-efficacy (Sanchez-Cardona et al., 2012). Research has found that agreeableness can lead to increased self-efficacy (Caprara et al., 2010). Certain researchers have found that individual self-efficacy is positively correlated with extraversion and negatively correlated with neuroticism (Schmitt, 2007). The finding of Djigić et al. (2014) indicated that conscientiousness predicts the self-efficacy of teachers, while Marcionetti and Rossier (2016) believed that conscientiousness, neuroticism, and extraversion are significantly correlated with self-efficacy. Furthermore, Brown and Cinamon (2016) proposed that higher conscientiousness and extraversion, and lower neuroticism, help enhance self-efficacy.

In sports, Wang et al. (2009) found that neuroticism has a significant negative predictive effect on the general self-efficacy of basketball players, while extraversion and conscientiousness have significant positive predictive effects. Based on the existing literature, the current study hypothesized that neuroticism is negatively correlated with self-efficacy, while agreeableness, conscientiousness, and extraversion are positively correlated with self-efficacy in boxers.

### Self-Control and Self-Efficacy

Self-efficacy may play a mediating role between personality and self-control. According to Bandura’s self-efficacy and self-regulation theories, self-control is affected by self-efficacy, and there was a significant positive correlation between the two (Bandura, 1978). Research found a positive correlation between self-efficacy and self-control (Au, 2015). Graham and Bray (2015) studied self-efficacy as a psychological factor to explain how self-control is performed. Baumeister’s analysis showed that self-control requires an individual’s own control resources, and self-efficacy complements this resource by acting as a positive emotion (Baumeister, 2002). Yu and Yang’s (2008) study concluded that there is an interaction effect between self-efficacy beliefs and self-control behavior.

A large number of researches have shown that there is a positive correlation between self-efficacy and self-control in different population groups (Huang and Yang, 2015). Jones et al. found that self-efficacy and sense of control are important indicators of an athlete’s state (Jones et al., 2009). Research shows that self-efficacy had a positive effect on self-control in athletes (Yang et al., 2013). Specifically, self-efficacy mediated the relationship between other psychological traits and self-control among different groups (Wang et al., 2009; Fang et al., 2015). Therefore, the present study further explores whether self-efficacy has a positive effect on self-control in boxers, and whether self-efficacy has a mediating effect between personality traits and self-control. The current study hypothesized: the self-efficacy and the self-control of Chinese boxers are positively correlated; the self-efficacy of Chinese boxers mediates the effects of neuroticism, agreeableness, conscientiousness, and extraversion on self-control.

## Materials and Methods

### Sample

The participants, acquired by cluster sampling, comprised boxers from Chinese national boxing teams as well as teams at several provinces and cities including Shenyang, Hubei, Anhui, Inner Mongolia, Chongqing, and Sichuan. All subjects gave written informed consent in accordance with the Declaration of Helsinki. Permission was obtained from the Southwest University’s Human Research Ethics Committee. Prior to answering the items, participants read information about the purpose of the study, implications of participation, and data protection. The information stressed that participation was completely voluntary and anonymous. A total of *N =* 230 questionnaires were distributed, and *N =* 210 valid ones (91%) were returned. Among the participants, *n =* 76 were males (36.2%) and *n =* 134 females (63.8%). There were 79 Level-3 athletes (37.6%), 24 Level-2 athletes (11.4%), 49 Level-1 athletes (23.3%), 45 Master-Level athletes (21.4%), and 13 athletes at the International Master-Level (6.2%). Level-3 is the lowest and the International Master-Level is the highest. The average age was *M =* 18.89 years (SD *=* 3.83), with the average prior training period being *M =* 4.93 years (SD *=* 3.22). Fifty participants were under 18 years of age and permissions from their parents were obtained; written and informed consent was obtained from the parents/legal guardians of all non-adult participants. The average time for completing the survey was 20 min.

### Procedure

After receiving informed consent from the management, coaches, and athletes of the national team and other sports teams, the questionnaires were distributed to teams at the provincial level or above. The instructions were explained in detail and example questions were provided to the participants, who were asked to read the questionnaire carefully and answer it. The questionnaires were distributed at Jilin Sport University, Wuhan Sport University, Anhui, Inner Mongolia, Chongqing, and Sichuan between the 11th of October and the 9th of November, 2017.

### Measures

#### NEO Five-Factor Inventory

Personality was measured with the validated Chinese NEO-PI-R (Costam and McCrae, 1992; Chen et al., 2016). The NEO-PI-R contains 60 items and is one of the most widely used measures for the Big Five personality traits. The self-report items are rated on a 5-point Likert scale ranging from “strongly disagree (1)” to “strongly agree (5)” and reflect the five higher order domains Neuroticism, Extraversion, Openness to Experiences, Agreeableness, and Conscientiousness. There are 12 items per FFM-FFI domain. A confirmatory factor analysis confirmed the one-dimensionality of the scale (CFA): *χ*^2^/*df* = 1.33, RMSE = 0.04, TLI = 0.99, GFI = 0.98, CFI = 0.99. The factor loadings of the items ranged between *a* = 0.50 and *a* = 0.73. The internal consistencies of the inventory’s five scales were *α* = 0.77 (Neuroticism), *α* = 0.76 (Agreeableness), *α* = 0.80 (Conscientiousness), *α* = 0.72 (Extraversion), and *α* = 0.57 (Openness). The Openness scale was not used furthermore due to its unsatisfactory internal consistency and its cultural unsuitability for Chinese boxers.

### Self-Control Questionnaire for Athletes

Self-control was measured by a 24-item, 5-point Likert Chinese scale questionnaire, ranging from “1 = not at all” to “5 = very much”; higher scores indicate better self-control (item example: “In order to complete the training task, I can endure extreme fatigue”). In a previous study, the scale provided a reference for athlete selection (Li and Zhang, 2011). A confirmatory factor analysis confirmed the one-dimensionality of the scale (CFA): *χ*^2^/*df* = 1.15, RMSEA = 0.03, TLI = 0.97, GFI = 0.92, NFI = 0.87, CFI = 0.98. The factor loadings of the items ranged from *a* = 0.49 to *a* = 0.75. The internal consistency of the questionnaire was good (*α* = 0.87).

### Self-Efficacy Scale for Athletes

Based on self-efficacy theory and related theories about competitive sports, Wei et al. (2008) compiled a self-efficacy scale for athletes in intensive sports. This scale consisted of 15 items, such as “I can keep my mind clear and focused during the competition.” Each item was measured by a 5-point scale (1 = never been like this; 5 = always so). A higher score indicates a higher self-efficacy. The one-dimensionality of the scale was proved by a confirmatory factor analysis (CFA): *χ*^2^/*df* = 1.07, RMSEA = 0.02, TLI = 0.99, GFI = 0.96, NFI = 0.95, CFI = 0.99, IFI = 0.99. The factor loadings of the items ranged from *a* = 0.43 to *a* = 0.75. The internal consistency of the scale was good (*α* = 0.92).

## Data Analyses

A two-way analysis of variance (ANOVA, LSD-*post hoc* test) was run for testing mean differences. The bias-corrected percentile bootstrap method was used to conduct regression analyses (Fang et al., 2012). To implement this method, we used the Model 4 PROCESS macro for SPSS created by Hayes (2013). Gender, age, years of training, and competitive level were controlled. The 95% confidence intervals of the mediating effects are reported. The statistical significance level was set to *α* = 0.05.

## Results

### Testing for Common Method Bias

To avoid response bias, some items in the questionnaires were expressed in reverse wording, AMOS 21.0 was used to conduct a CFA, with the common factor of all variables set to 1, and all item variables were used as explicit variables. The CFA results showed that the model fit was low, indicating no serious common method bias. (*χ*^2^/*df* = 2.01, RMSEA = 0.07, NFI = 0.34, CFI = 0.50, TLI = 0.49, GFI = 0.55, IFI = 0.50).

### Self-Control and Self-Efficacy: Group Differences

The averaged item score of the self-control was *M =* 3.68 (SD = 0.49), indicating a relatively high level of self-control among boxers in China. This study also examined the effect of gender and competitive level differences on self-control; the results indicated no significant gender differences (*F* = 1.14, *p* = 0.28, *d* = −0.011), but a significant main effect of competitive level (*F* = 7.81, *p* < 0.01, *η*^2^ = 0.12). The interaction between gender and competitive level was not significant (*F* = 1.82, *p* = 0.13, *η*^2^ = 0.04). The item-based averaged self-control scores of boxers from the five different competitive levels were significantly different. The higher the competitive level, the higher the level of self-control (International Master-Level: *M =* 3.92, SD = 0.62; Master-Level *M =* 3.79, SD = 0.48; Level-1: *M =* 3.77, SD = 0.45, Level-2: *M =* 3.83, SD = 0.49; Level-3: *M =* 3.47, SD = 0.43. The simple analysis showed that the averaged item score of self-control in International Master-Level was significantly higher than that of the Level-3, *p* < 0.01, *d* = 0.98).

The average item score of self-efficacy was *M =* 3.50 (SD = 0.64), indicating that the Chinese boxers’ self-efficacy exceeds the theoretical item mean. There was no significant difference between male and female boxers (*p* > 0.05, *d* = 0.24). The mean item scores of self-efficacy among boxers from five different competitive levels differed significantly: the higher the competitive level, the higher the self-efficacy (International Master-Level: *M =* 3.81, SD = 0.76; Master-Level: *M =* 3.66, SD = 0.60; Level-1: *M =* 3.53, SD = 0.58; Level-2: *M =* 3.60, SD = 0.71; Level-3: *M =* 3.30, SD = 0.60). There was a significant difference on self-efficacy between International Master-Level and Level-3 (*p* < 0.01, *d* = 0.81).

### Personality Traits, Self-Efficacy, and Self-Control: Correlations

Neuroticism was significantly and negatively correlated with self-efficacy and self-control, while extraversion, agreeableness, and conscientiousness were significantly and positively correlated with self-efficacy and self-control. Self-efficacy and self-control were positively correlated (see Table 1).

**Table 1:** Means, standard deviations, and correlation coefficients of personality traits, self-control, and self-efficacy.

	*M*	SD	1	2	3	4	5	6
Neuroticism	2.94	0.53	1					
Extraversion	3.63	0.49	−0.40^*^	1				
Agreeableness	3.78	0.46	−0.49^*^	0.37^*^	1			
Conscientiousness	3.50	0.48	−0.52^*^	0.47^*^	0.54^*^	1		
Self-efficacy	3.50	0.64	−0.42^*^	0.48^*^	0.45^*^	0.70^*^	1	
Self-control	3.68	0.49	−0.57^*^	0.48^*^	0.68^*^	0.74^*^	0.70^*^	1

* *Indicates p < 0.05*.

### Testing for Mediation by Self-Efficacy on Effects of Neuroticism, Agreeableness, Extraversion, and Conscientiousness on Self-Control

This study used the Bootstrap method proposed by Fang et al. (2012) and the Model 4 PROCESS macro for SPSS created by Hayes (2013) to conduct mediating effect testing; gender, competitive level, age, and years of training were set as control variables.

Regression analysis showed that neuroticism negatively predicted self-efficacy (*β* = −0.23, *p* < 0.01), while self-efficacy positively predicted self-control (*β* = 0.88, *p* < 0.001). Neuroticism negatively predicted self-control (*β* = −0.32, *p* < 0.001). Extraversion was a positive predictor of self-efficacy (*β* = 0.17, *p* < 0.001), while self-efficacy positively predicted self-control (*β* = 0.78, *p* < 0.001). Extraversion and self-efficacy were positive predictors of self-control (*β* = 0.27, *p* < 0.001). Agreeableness positively predicted self-efficacy (*β* = 0.26, *p* < 0.001), and self-efficacy was a positive predictor of self-control (*β* = 0.77, *p* < 0.001), as was agreeableness (*β* = 0.44, *p* < 0.001). Conscientiousness positively predicted self-efficacy (*β* = 0.43, *p* < 0.001), and self-efficacy was a positive predictor of self-control (*β* = 0.58, *p* < 0.001), as was conscientiousness (*β* = 0.47, *p* < 0.001).

**Figure 1:**
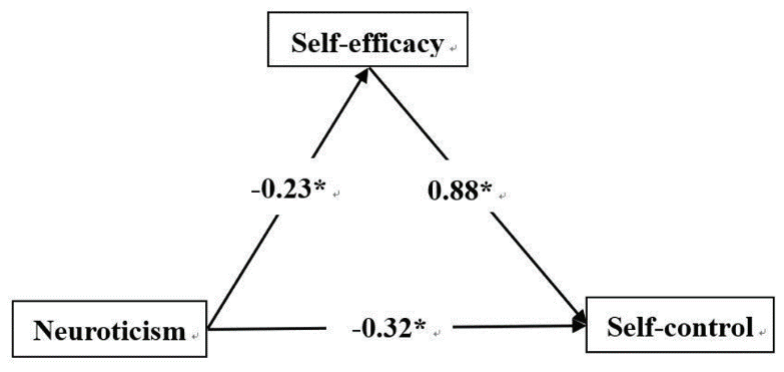
Neuroticism → self-efficacy → self-control. *Statistically significant.

**Figure 2:**
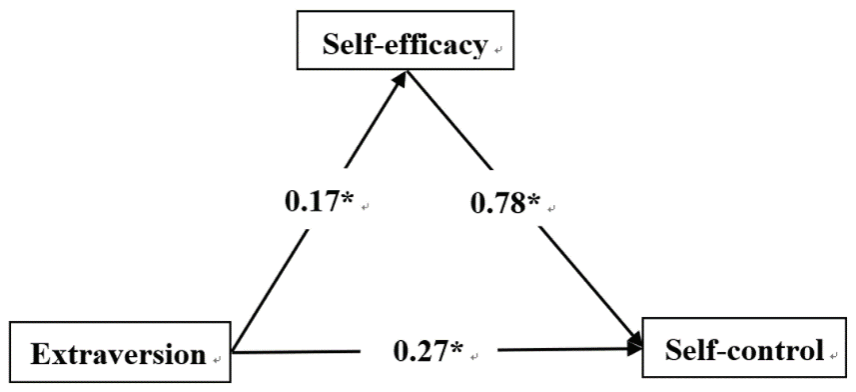
Extraversion → self-efficacy → self-control. *Statistically significant.

**Figure 3:**
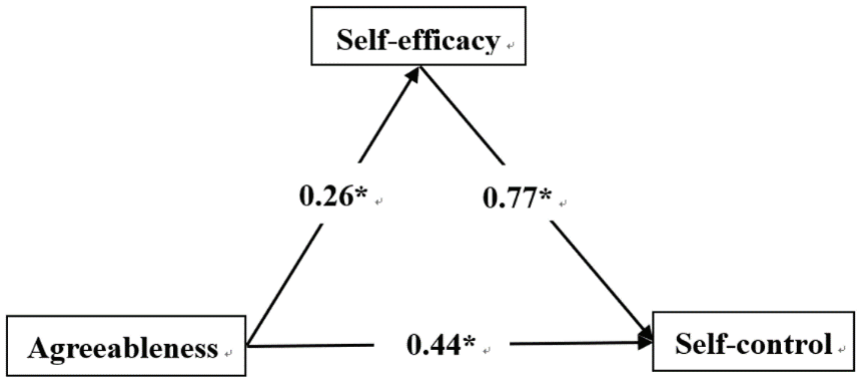
Agreeableness → self-efficacy → self-control. *Statistically significant.

**Figure 4:**
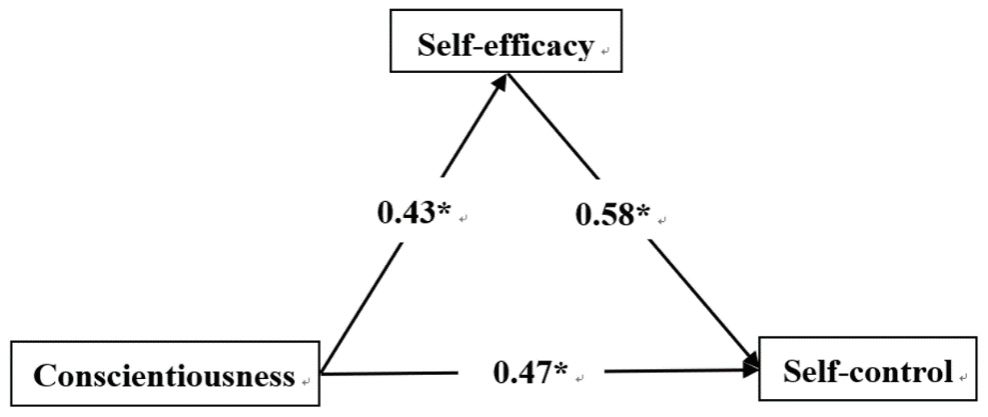
Conscientiousness → self-efficacy → self-control. *Statistically significant.

Figures 1–4 present the standardized effects of the paths of neuroticism, extraversion, agreeableness, and conscientiousness on self-control. The bootstrap 95% confidence interval of the indirect effect of self-efficacy did not comprise 0, indicating that self-efficacy had a significant mediating effect on neuroticism, extraversion, and agreeableness. Conscientiousness had a mediating effect on self-control. Taking together, neuroticism explained 22% of the change in self-efficacy, extraversion 27%, agreeableness 26%, and conscientiousness 50%. As such, H_4_ was confirmed.

## Discussion

### Direct Influence of Personality Traits on Self-Control

In the current study, there were significant positive correlations between agreeableness, conscientiousness, extraversion, and self-control, and a negative correlation between neuroticism and self-control.

Previous studies have shown that agreeableness, conscientiousness, and extraversion are positively related to self-control (Bazzy et al., 2017), while neuroticism significantly correlated with self-control (Andrei et al., 2014). Based on those studies, the current study assumed that neuroticism is negatively correlated with self-control, while agreeableness, conscientiousness, and extraversion are positively correlated with self-control. These hypotheses are supported and are consistent with the results of Deng and Gao (2015) and Pilarska (2018).

Avoiding injuries and reducing the number of fouls are very important skills for boxers. Self-control is a very important psychological characteristic that influences those skills (Yu et al., 2012). By understanding the mediating relationship between personality and self-control, boxers can not only avoid injuries but also reduce the number of fouls by improving their self-control level and their own skills and tactics (Ba and Zhu, 2008). Song et al. (2009) suggested that boxers should continuously enhance their psychological stability by focusing on the actual combat element of boxing. Therefore, the Big Five factors and the self-control could perhaps be used as psychological criteria to select boxers and the relationship between them is an important first step in order to further boxers. Longitudinal research is needed to evaluate the relevance of these predictors.

### The Mediating Role of Self-Efficacy Between Personality Traits and Self-Control

Studies have shown that four of the Big Five factors have indirect predictive effects on self-control through self-efficacy. Theories of self-efficacy hold that the subjective judgment and self-feeling that individuals have about their ability to perform a certain behavior or tasks play an important role in the decision-making process before a behavior is performed (Eastin and La-Rose, 2000). In sporting contexts, scholars have found that the stronger an athletes’ self-efficacy, the better their performance in sports as diverse as athletics, tennis, scuba diving, and gymnastics (Vancouver et al., 2002): when athletes perform a task for the first time, their self-efficacy affects their performance (Myers et al., 2004). Other studies have demonstrated that, even despite excellent skills and motivation to win, people with outstanding capabilities are less likely to achieve success if they have poor self-efficacy (Earley and Lituchy, 1991). The present study hypothesized that neuroticism is negatively correlated with self-control, while agreeableness, conscientiousness, and extraversion are positively correlated with self-control; and these hypotheses were supported by the results of this study and are consistent with the results of Zhang (2016) and Marcionetti and Rossier (2016). Moreover, the recent study found that self-efficacy has a significant positive predictive effect on self-control, consistent with the results of Huang and Yang (2015) and Kaida et al. (2006).

The present study also hypothesized that self-efficacy mediates the effects of neuroticism, agreeableness, conscientiousness, and extraversion on self-control. The present study observed four mediating paths, showing partial mediating effects of self-efficacy on self-control for each personality trait variable. Many other researchers have also found that self-efficacy mediates the link between personality traits and self-control, such as Fang et al. (2015) and Stajkovic et al. (2018). Therefore, cultivating and upgrading the self-efficacy of boxers should be emphasized in their training.

## Limitations

Firstly, with the cross-sectional design, no causal relationship could be established. Secondly, the present study focused on the role of self-efficacy in the relationship between Big Five personality traits and self-control, but there are still many other important mediating variables which may influence self-control, such as depression, anxiety, or self-esteem and self-concept, which should also be explored in future research. Finally, the current study focused on Chinese boxers. Further research is needed to figure out whether the results of this study can be generalized to other sports or western cultures.

Despite these limitations, the present study contributes to understand the inherent relationship between big five personality and self-control as well as its possible mechanisms among Chinese boxers. The exploration of the variables can add a fruitful avenue toward the development of knowledge about the processes involved in self-control in sport.

## Conclusions

First, there were significant differences in self-control and self-efficacy among boxers of different competitive levels. Second, there are significant correlations of neuroticism and agreeableness, conscientiousness, and extraversion with self-control, indicating that these four dimensions are direct statistical predictors of self-control. Self-efficacy is positively correlated with self-control. Finally, boxers’ self-efficacy mediates personality traits and self-control, indicating that personality traits predict self-control not only directly but also indirectly through self-efficacy. Overall, the mediation effect models constructed in this study had some explanatory power for self-control.

## Data Availability

The raw data supporting the conclusions of this manuscript will be made available by the authors, without undue reservation, to any qualified researcher.

## Ethics Statement

The protocol was approved by the Southwest University’s Human Research Ethics Committee. Prior to initiation of the study, all subjects gave written informed consent in accordance with the Declaration of Helsinki.

## Author Contributions

All authors listed have made a substantial, direct and intellectual contribution to the work, and approved it for publication.

### Conflict of Interest Statement

The authors declare that the research was conducted in the absence of any commercial or financial relationships that could be construed as a potential conflict of interest.
